# The role of migration in mental healthcare: treatment satisfaction and utilization

**DOI:** 10.1186/s12888-022-03722-8

**Published:** 2022-02-15

**Authors:** Gabriele Gaigl, Esther Täumer, Andreas Allgöwer, Thomas Becker, Johanna Breilmann, Peter Falkai, Uta Gühne, Reinhold Kilian, Steffi G. Riedel-Heller, Klemens Ajayi, Jessica Baumgärtner, Peter Brieger, Karel Frasch, Stephan Heres, Markus Jäger, Andreas Küthmann, Albert Putzhammer, Bertram Schneeweiß, Michael Schwarz, Markus Kösters, Alkomiet Hasan

**Affiliations:** 1 Department of Psychiatry and Psychotherapy, University Hospital, Klinikum der Universität München, Ludwig-Maximilians University Munich, Nußbaumstraße 7, D-80336 Munich, Germany; 2 Institute for Epidemiology and Medical Biometry, Ulm University, Ulm, Germany; 3 Department of Psychiatry II, Ulm University, BKH, Günzburg, Germany; 4 Institute of Social Medicine, Occupational Health and Public Health (ISAP), Medical Faculty, University of Leipzig, Leipzig, Germany; 5 Kbo-Isar-Amper hospital, Region Munich, Germany; 6 Department of Psychiatry, Psychotherapy and Psychosomatic, University of Augsburg, Medical Faculty, BKH Augsburg, Augsburg, Germany; 7 District hospital Donauwörth, Donauwörth, Germany; 8 District hospital Kempten, Kempten, Germany; 9 District hospital Memmingen, Memmingen, Germany; 10 District hospital Kaufbeuren, Kaufbeuren, Germany

**Keywords:** Immigration, Mental healthcare, Patient satisfaction, Service use, Patient needs

## Abstract

Migration rates increase globally and require an adaption of national mental health services to the needs of persons with migration background. Therefore, we aimed to identify differences between persons with and without migratory background regarding (1) treatment satisfaction, (2) needed and received mental healthcare and (3) utilization of mental healthcare.

In the context of a cross-sectional multicenter study, inpatients and day hospital patients of psychiatric settings in Southern Germany with severe affective and non-affective psychoses were included. Patients’ satisfaction with and their use of mental healthcare services were assessed by VSSS-54 and CSSRI-EU; patients’ needs were measured via CAN-EU.

In total, 387 participants (migratory background: *n* = 72; 19%) provided sufficient responses for analyses. Migrant patients were more satisfied with the overall treatment in the past year compared to non-migrant patients. No differences between both groups were identified in met and unmet treatment needs and use of supply services (psychiatric, psychotherapeutic, and psychosocial treatment).

Despite a comparable degree of met and unmet treatment needs and mental health service use among migrants and non-migrants, patients with migration background showed higher overall treatment satisfaction compared to non-migrants. The role of sociocultural and migrant-related factors may explain our findings.

**Supplementary Information:**

The online version contains supplementary material available at 10.1186/s12888-022-03722-8.

## Background

The number of persons with migration background^1^ is increasing due to socio-political, economic, demographic and environmental factors [2, 3]. In 2019, the population with migration background in Germany comprised 26% of the population in total [1]. Even though more than 99% of migrants living in Germany exhibit a health insurance [4], which covers on average of 84% of healthcare costs [5], there is still an underrepresentation of people with migratory background in the German healthcare system [6–9]. Despite the higher psychopathological burden of people with migration background [4], recent findings indicate an underrepresentation of 1st generation migrants in the German in- and outpatient mental healthcare system [7, 8], including the psychosocial supply system [9]. Moreover, migration factor was shown to be a negative predictor for the treatment outcome of mental disorders [10]. Turkish descents, representing the major group of immigrants in Germany, were found to exhibit inferior effective treatment results after receiving psychosomatic rehabilitative treatment compared to patients without migration background [11]. Furthermore, a number of studies have found that patients, belonging to an ethnic minority, are less likely to engender an empathic response from their clinicians, to participate in shared decision making and to receive information about disorder and treatment compared to ethnic majorities [12]. One could assume that these research findings are associated with lower treatment satisfaction. Likewise, a recent review on patient satisfaction with psychiatric inpatient services indeed indicates a positive relationship of treatment satisfaction with treatment outcome, quality of the therapeutic relationship as well as information sharing [13]. However, results on treatment satisfaction among migrants remain widely inconsistent and are still sparse in the field of mental healthcare [13–15]. Numerous definitions of patient satisfaction exist [16]. Yet, the majority of definitions capture the following aspects: patient satisfaction as a correspondence between patients’ needs or expectations and their actual experiences with healthcare services [14, 16, 17]. In the evaluation of service quality, patient satisfaction plays a key role, as it represents the unique perspective of patients and the renunciation of a clinicians’ centered view. Shipley and colleagues (2000) [18] demonstrated that patient satisfaction was a more accurate indicator of quality of care than clinicians’ evaluation of the treatment. Moreover, patient satisfaction was found to improve adherence to treatment, which is crucial in the context of relapse and recurrence prevention in severe mental disorders, like affective and non-affective psychoses [19]. Patients’ needs, expectations, treatment experiences and thus satisfaction are indicated to be influenced by a conglomerate of cognitive-affective (e.g., knowledge about care, prior experiences, values, cultural norms) as well as sociodemographic factors (e.g., age, socio-economic status, geographic characteristics) [13, 14, 20, 21]. On the one hand, the complexity of the concept treatment satisfaction requires interpreting results against the background of possible moderators. On the other hand, it highlights the relevance of examining treatment satisfaction in an ethnically and culturally diverse society with a high percentage of migrants, such as Germany [16].

Investigating migration-related disparities is generally a difficult endeavor not least due to the heterogeneity that is inherent in migration (e.g., country of origin, reason for migration) [22]. However, the indicated disadvantages of people with migration background in the mental health care system [6–8, 10] as well as the lack of representative research [8] require comparative studies to raise understanding of possible underlying factors. Thus, our aim was to investigate the quantity and quality of treatment among patients with and without migration background in the framework of a multicenter study by examining the following aspects: (1) treatment satisfaction as indicator of quality of care [18], (2) the degree of accordance between needed and actually received mental healthcare as aspect of patient satisfaction and thus quality of care [13, 18], and (3) utilization of mental healthcare services as indicator of treatment quantity.

## Design and methods

### Subjects and recruitment

The cross-sectional study was performed from 03/2019 to 09/2019 in the context of a larger project (Implementation status of the German guideline for psychosocial interventions for patients with severe mental illness (IMPPETUS)) [23]. Inpatients and day hospital patients of psychiatric settings diagnosed with severe affective and non-affective psychoses were included. As we conducted a multi-centric study, data was collected in 10 departments for psychiatry and psychotherapy in Bavaria, Germany. These departments are characterized by providing both psychiatric (i.e. somatic treatment forms, e.g., pharmacotherapy) and psychotherapeutic forms of treatment (e.g., cognitive and behavioral therapy). The selected centers represent metropolitan (Munich, Augsburg), middle-urban (Kempten, Memmingen) as well as rural (Donauwörth, Günzburg, Kaufbeuren, Taufkirchen) catchment areas (the list of the participating hospitals appears in the supplement).

**Fig. 1:**
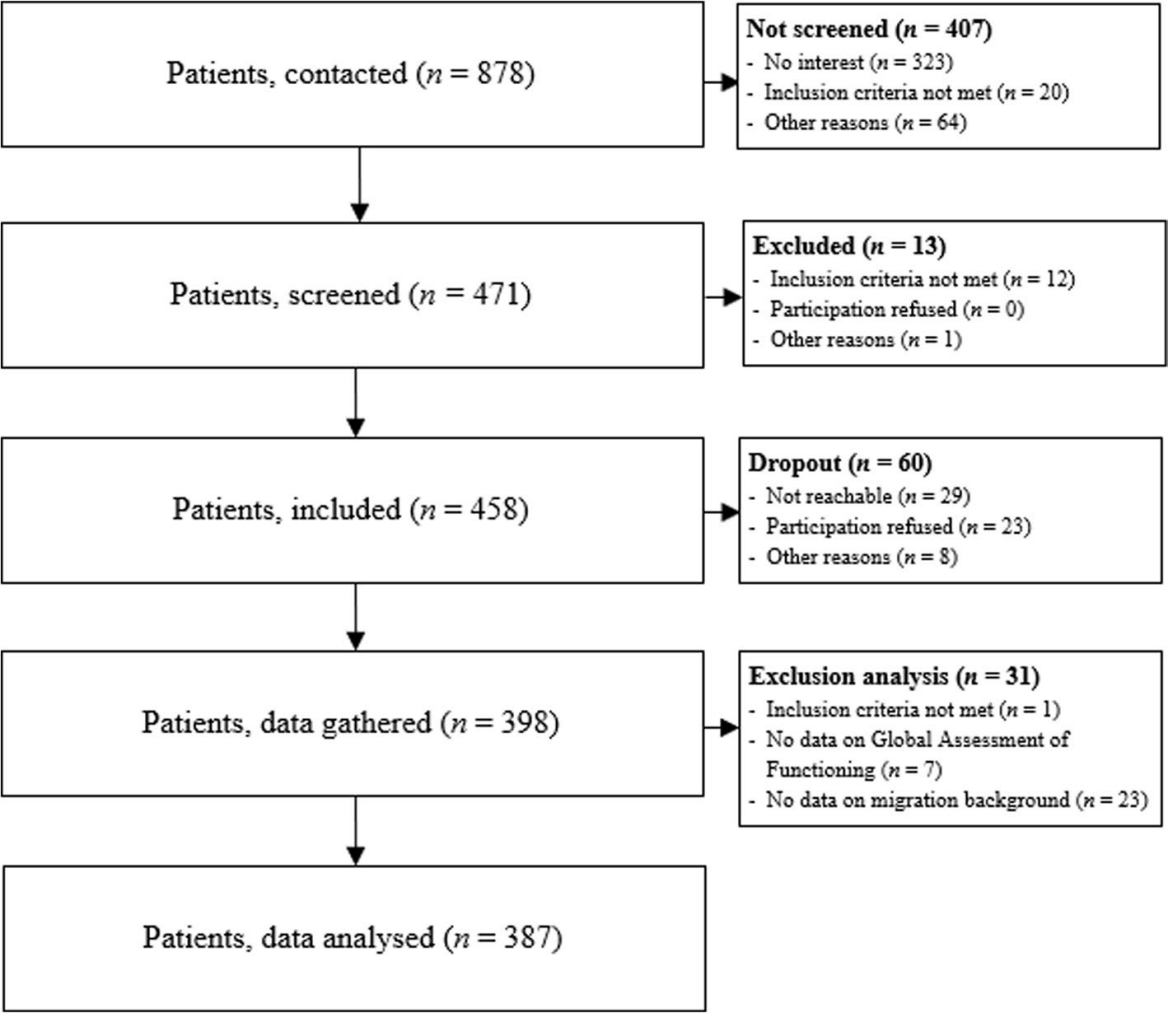
Recruitment and study flow chart. N = number of participants

In the present study, the following inclusion criteria were applied: (1) 18 to 65 years old, (2) ability to give consent, (3) sufficient German language skills in order to understand the questions exclusively asked in German, (4) exhibiting a severe mental illness. In order to identify patients with severe mental illness, the subsequent criteria were used [24]: (a) Patients with schizophrenia (ICD-10: F2), bipolar disorders (F30, F31) or depression (F32, F33), (b) duration of psychiatric illness ≥2 years, (c) considerable consequences on daily life activities and social functioning, which was assessed through the Global Assessment of Functioning, GAF, [25] (score ≤ 60) and Health of the Nation Outcome Scales, HoNOS, [26] (score of ≥2 on one of the subscale items for symptomatic problems and a score of ≥2 on each, or a score of ≥3 on at least one of the four subscale items for social problems). The recruitment and study flow chart is displayed in Fig. 1.

Trained study personnel invited each eligible patient (during their attendance at the clinic) to participate in the study in coordination with the clinical teams. Those who agreed to participate were screened by trained study staff as soon as possible after admission. To identify patients with severe mental disorder, the Global Assessment of Functioning (GAF) [25] and the Health of the Nation Outcome Scales (HoNOS) [26] were executed. The diagnosis was set by the treating board-certified psychiatrist in the beginning of the inpatient or day hospital treatment. The duration of the illness (criterion: ≥ 2 years) was taken from the medical record or from the information provided by the treating physician. Patients who met the inclusion criteria were interviewed by trained study personnel shortly (ca. 2 weeks) before their discharge. The research team was informed by the treating physician about the (approximate) date of discharge to interview participants in case of a premature, prolonged, and planned discharge. There were no restrictions regarding the time period between inclusion in the study (shortly after admission) and conducting the interview (shortly before discharge).

### Measures

After recruitment of patients who met the inclusion criteria, the following constructs were measured shortly (ca. 2 weeks) before discharge from the clinic.

#### Sociodemographic data

To assess sociodemographic aspects (including migration background) we used the German adaptation of the Client Sociodemographic and Service Receipt Inventory (CSSRI-EU) [27]. Migration background was measured through the following item: “Do you have a migration background?”. In case of agreement, participants were asked to specify their migration background: “Yes, I am a migrant myself.” (1st generation migrant) vs. “Yes, at least one of my parents is a migrant.” (2nd generation migrant). As detailed below, we focused on the analysis of migrants (1st and 2nd generation) vs. non-migrants. The rationale for this classification was both content-based and statistical. The reason to group 1st and 2nd generation migrants in one category is that they both exhibit direct or – in case of 2nd generation migrants - indirect migration experience. Furthermore, against the backdrop of statistical power, the undertaken categorization reduced the chance to create a type II error - alternative distributions (undertaken for sensitivity analyses) would have led to a greater inequality between the compared groups as well as to smaller subgroup sizes [28].

Moreover, we conducted two exploratory sensitivity analyses: 1st generation vs. 2nd generation vs. non-migrants; non-migrants and 2nd generation migrants vs. 1st generation migrants.

#### Service satisfaction and utilization

##### Service satisfaction

In order to assess patients’ satisfaction with mental healthcare services in the previous year, we conducted the German adaptation of the Verona Service Satisfaction Scale (VSSS-54) [29]. Psychometric properties of the German adaptation are satisfying and comparable with international studies [29, 30]. Conceptually, the 54 items of the VSSS-54 represent seven dimensions: *Overall Satisfaction* (three items: satisfaction with the amount of help received, the kind of treatment services, the overall treatment services), *Professionals’ Skills and Behavior* (24 items: satisfaction with professionals’ behavior, e.g. interpersonal skills), *Information* (three items: satisfaction with information on disorders, therapies and services), *Access* (two items: satisfaction with service location and costs), *Efficacy* (eight items: satisfaction with overall and specific aspects of efficacy of service, e.g. social skills), *Relatives Involvement* (six items: satisfaction with help given to relatives/persons of trust) and *Types of Intervention* (17 items: satisfaction with and use of e.g. medical prescription, psychotherapy). The items of the VSSS-54 dimensions were rated on a 5-point Likert scale (level of satisfaction: 1 = terrible - 5 = excellent). Further methodological information is presented in Table 2 and Supplementary Methods.

##### Service utilization

The VSSS-54 domain *Types of Intervention* was analyzed on a (binary) item-based level to assess the utilization of specific mental healthcare services within the past 12 months before the survey: medical prescription and psychotherapy (individual and family therapy). Moreover, the receipt of outpatient psychiatric and psychotherapeutic treatment 3 months before admission to the hospital (binary items) was assessed through the German adaptation of the CSSRI-EU [27]. The use of psychosocial interventions was surveyed via the questionnaire “Attitudes and knowledge regarding psychosocial therapies” developed by the authors and available on request. The utilization of the presented psychosocial therapy forms was analyzed through binary items on (e.g., question: “Have you ever received supported employment?”). The psychosocial supply system in general and psychosocial interventions in particular focus on improving the individual’s integration and participation in society through individual psychosocial interventions (e.g., occupational therapy, exercise therapy), system-level interventions (e.g., residential care interventions), and cross-cutting interventions (e.g., peer-led interventions) [31]. We analyzed whether the participants have ever received specific forms of psychosocial interventions (system-level interventions, single psychosocial interventions and cross-cutting issues). See Supplementary Methods for further methodological information.

#### Patient needs

The German adaptation of the Camberwell Assessment of Need-EU (CAN-EU) [32] was applied to measure the extent of accordance between needed and actually received mental healthcare. The interviewer-administered instrument consists of the following five categories of need: *Basic*, *Functioning, Health, Social* and *Services*. Information on the individual domains is presented in Table 4 and Supplementary Methods. The participants were asked whether there was a need regarding the individual domains in the past 4 weeks. In case of an absence of need/problem, the interviewer proceeded with the next domain. In case of a need, the participant was asked whether adequate care was received. If the subject agreed, it was recorded as *met need*, in case of disagreement (no or inadequate care received), it was registered as *unmet need*. For our study, we computed two summary scores: the total number of met needs (one or more met needs but no unmet needs on the domains within the category) as well as the total number of unmet needs (at least one unmet need on the domains belonging to the category) [33]. Concerning internal consistency, test-retest-reliability and inter-rater-reliability, the German version of the CAN-EU is satisfying and comparable with other European versions [32, 34].

### Statistical analysis

All analyses were carried out in IBM SPSS for Windows (version 26) with a significance level of α = 0.05. Descriptive statistics are displayed with frequency and percentage distributions for binary data. Means and standard deviations are presented in case of continuous data and additionally medians for categorical data. Intergroup differences were assessed using Chi^2^ tests in case of binary data and Mann-Whitney-U or Kruskal-Wallis tests (Dunn-Bonferroni tests for subgroup analyses in case of significant intergroup differences) for categorical data (e.g., in the event of patient’s satisfaction, assessed by 5-point Likert scales). For continuous data we used independent sample t-tests or one-way ANOVAs (Bonferroni tests for subgroup analyses in case of significant intergroup differences). As primary intergroup analyses, we compared participants with vs. without migratory background (1st and 2nd generation migrants). As exploratory secondary analyses we conducted two sensitivity analyses: First, we computed a 3-group-comparison to identify differences between 1st generation migrants, 2nd generation migrants and subjects without migratory background. Second, we analyzed differences between native Germans (subjects without migratory background and 2nd generation migrants) and 1st generation migrants.

## Results

### Participants’ characteristics

In total, 398 patients participated in the study. Retrospectively, *n* = 8 patients were excluded from the analyses (of which *n* = 7 did not provide data on the Global Assessment of Functioning, and *n* = 1 did not fulfill the age inclusion criteria). Moreover, only participants who provided responses to their migration status were included in the analyses (*N* = 387, of which *n* = 72 exhibited a migration background). Demographic information of the subjects is described in Table 1. Results indicate that subjects with migratory background in our sample were more frequent to exhibit schizophrenia (ICD-10: F2x), *p* = 0.031, and lived more often in medium populated areas (20′001–500′000 inhabitants), *p* = 0.007, compared to non-migrant subjects. Moreover, participants without migratory background were more frequently located in lower populated areas (≤ 20′000 inhabitants), *p* = 0.001, showed a higher functioning level, *p* = 0.008 and a lower severity of mental disorder, *p* = 0.037 than migrant participants. No intergroup differences were found concerning age, gender, family status and salary (net). For all details and complete test statistics see Table 1 and Supplementary Tables 1 and 5 (secondary analyses).

**Table 1:** Descriptive statistics and mean response comparisons between patients with and without migratory background

	Total	No migration background		Migration background ^**a**^		Test Statistics		
	n (% Yes)	N	n (% Yes)	N	n (% Yes)	X^**2**^	df	p
**Gender** (*N* = 387)								
Female	220 (56.8%)	315	178 (56.5%)	72	42 (58.3%)	0.08	1	0.778
**Diagnosis** ^**b**^ (*N* = 387)								
Schizophrenia	125 (32.3%)	315	94 (29.8%)	72	31 (43.1%)	4.68	1	**0.031**
Depression	225 (58.1%)	315	187 (59.4%)	72	38 (52.8%)	1.05	1	0.307
Bipolar Disorder	37 (9.6%)	315	34 (10.8%)	72	3 (4.2%)	2.98	1	0.084
**Family status** (*N* = 387)								
Single	217 (56.1%)	315	185 (58.7%)	72	32 (44.4%)	6.70	3	0.082
Married	88 (22.7%)	315	65 (20.6%)	72	23 (31.9%)			
Divorced	69 (17.8%)	315	56 (17.8%)	72	13 (18.1%)			
Widowed	13 (3.4%)	315	9 (2.9%)	72	4 (5.6%)			
**Population size** (*N* = 386)								
≤ 20,000	160 (41.5%)	314	143 (45.5%)	72	17 (23.6%)	11.61	1	**0.001**
20,001–500,000	125 (32.4%)	314	92 (29.3%)	72	33 (45.8%)	7.31	1	**0.007**
> 500,000	101 (26.2%)	314	79 (25.2%)	72	22 (30.6%)	0.883	1	0.347
	**M (SD)**	**N**	**M (SD)**	**N**	**M (SD)**	**t**	**df**	**p**
**Age** (*N* = 385)								
Years	42.84 (13.08)	313	43.16 (13.14)	72	41.49 (12.87)	0.98	383	0.329
**Salary** (*N* = 151)								
Euro, net	1652.57 (1116.96)	130	1686.37 (1125.65)	21	1443.33 (1063.41)	0.93	149	0.357
**GAF** ^**c**^ (*N* = 387)								
	42.29 (9.78)	315	42.92 (9.91)	72	39.54 (8.73)	2.67	385	**0.008**
**HoNOS** ^**d**^ (*N* = 387)								
	22.32 (5.95)	315	22.03 (5.97)	72	23.61 (5.74)	−2.05	385	**0.042**

^a^Includes first- or second-generation migrants (as for all following tables in the manuscript)

^b^The diagnosis assignment was based on the ICD-10 classification system: F2x (schizophrenia), F32, F33 (depression), F30, F31 (bipolar disorder)

^c^Global Assessment of Functioning: higher values indicate a higher level of functioning

^d^Health of the Nation Outcome Scales: higher values indicate a higher severity of mental disorder

*N, n* Number of participants, *M* Means, *SD* Standard deviations, *X*^*2*^ Chi^2^-value, *t* t-statistics, *df* degrees of freedom

### Service satisfaction and utilization

Concerning satisfaction with mental healthcare services in the previous year (VSSS-EU), Mann-Whitney-U tests indicate a higher *Overall Satisfaction* with mental health care services (*p* = 0.006) as well as satisfaction with *Relatives Involvement* (*p* = 0.001) among patients with migratory background compared to non-migrants (Table 2). The number of intergroup differences increased when comparing 1st generation migrants with participants born in Germany. 1st generation migrants exhibited higher degrees of satisfaction compared to the reference group (no or 2nd generation migrants) in the following domains: *Overall Satisfaction, Professionals’ Skills and Behavior, Efficacy* and *Relatives Involvement*, all *p*-values ≤0.043 (see Supplementary Table 6).

**Table 2:** Treatment satisfaction: Average confirmation rates of the VSSS-EU dimensions and response comparisons

	Total				No migration background				Migration background				MWU Test		
	N	Mdn	M	SD	N	Mdn	M	SD	N	Mdn	M	SD	U	Z	p
**Overall Satisfaction**	366	4.00	3.78	0.81	298	3.67	3.72	0.80	68	4.00	4.01	0.78	8005.00	−2.73	**0.006**
**Professionals’ Skills and Behavior**	169	4.00	3.93	0.69	144	4.00	3.88	0.71	25	4.06	4.19	0.50	1376.00	−1.88	0.060
**Information**	346	3.67	3.41	0.95	285	3.67	3.38	0.95	61	3.67	3.58	0.90	7615.50	−1.53	0.126
**Access**	341	4.00	3.61	0.92	276	3.50	3.57	0.92	65	4.00	3.78	0.91	7833.50	−1.61	0.107
**Efficacy**	167	3.50	3.35	0.92	140	3.50	3.29	0.90	27	3.88	3.69	0.94	1474.50	−1.81	0.071
**Relatives Involvement**	147	3.60	3.33	1.15	125	3.40	3.21	1.14	22	4.20	3.99	1.01	777.00	−3.25	**0.001**

The items of the presented dimensions were rated on a 5-point Likert scale (level of satisfaction: 1 = terrible to 5 = excellent). *N, n* Number of participants, *Mdn* Medians, *M* Means, *SD* Standard deviations, *MWU* Mann-Whitney-U Test, *U* U-value, *Z* Standard score

Concerning the use of mental healthcare services, no differences between migrants and non-migrants were found in the examined areas of treatment: medical prescription and psychotherapy in the previous year, outpatient psychological-psychotherapeutic and medical-psychiatric treatments 3 months before admission to the hospital and psychosocial interventions. For complete test statistics see Table 3 and Supplementary Tables 3 and 7 (secondary analyses).

**Table 3:** Service utilization: Average confirmation rates of treatment use and response comparisons

	Total			No migration background			Migration background			Chi^**2**^ Test		
	N	n (% Yes)		N	n (% Yes)		N	n (% Yes)		X^**2**^	df	p
**Medical treatment**												
Medication prescription ^a^	360	353 (98.1%)		294	290 (98.6%)		66	63 (95.5%)		2.87	1	0.090
Outpatient medical-psychiatric treatment ^b^	383	151 (39.4%)		312	127 (40.7%)		71	24 (33.8%)		1.15	1	0.283
**Psychotherapeutic treatment**												
Individual psychotherapy ^a^	370	254 (68.6%)		301	210 (69.8%)		69	44 (63.8%)		0.94	1	0.333
Family therapy ^a^	366	73 (19.9%)		299	64 (21.4%)		67	9 (13.4%)		2.18	1	0.140
Outpatient psychological-psychotherapeutic treatment ^b^	384	89 (23.2%)		312	72 (23.1%)		72	17 (23.6%)		0.01	1	0.923
	**N**	**Mdn**	**M (SD)**	**N**	**Mdn**	**M (SD)**	**N**	**Mdn**	**M (SD)**	**MWU Test**		
										**U**	**Z**	**p**
**Psychosocial treatment**												
Score ^c^	384	0.28	0.32 (0.17)	314	0.28	0.32 (0.17)	70	0.24	0.28 (0.15)	9839.50	−1.38	0.169

^a^Participants were asked whether they had received the intervention in the last 12 months (VSSS-EU). The related time period of 12 months refers to the inpatient setting (at the time of the survey) and previous settings (out- or inpatient settings)

^b^Participants were asked whether they had received the intervention 3 months before admission to clinic (CSSRI-EU)

^c^Participants were asked whether they had ever received the psychosocial intervention. The displayed score corresponds to the proportion of psychosocial interventions used out of the total number of psychosocial interventions presented, shown as decimal number

*N, n* Number of participants, *X*^*2*^ Chi^2^-value, *df* degrees of freedom, *Mdn* Medians, *M* Means, *SD* Standard deviations, *MWU* Mann-Whitney-U Test, *U* U-value, *Z* Standard score

### Patient needs

Across all domains of need of the CAN-EU, we identified a higher percentage of unmet compared to met needs, see Table 4. Chi^2^ tests of independence showed no intergroup differences in the met and unmet needs of the CAN-EU dimensions between patients with vs. without migration background (Table 4). Moreover, secondary analyses on patients’ needs indicate no differences between groups, see Supplementary Tables 4 and 8.

**Table 4:** Met and unmet needs: Average confirmation rates of the CAN-EU-dimensions of need and response comparisons

	Total	No migration background		Migration background		Chi^**2**^ Test		
	n (% Yes)	N	n (% Yes)	N	n (% Yes)	X^**2**^	df	p
**Basic** ^**a**^ (*N* = 378)								
Met needs	96 (25.4%)	308	84 (27.3%)	70	12 (17.1%)	3.09	1	0.079
Unmet needs	149 (39.4%)	308	115 (37.3%)	70	34 (48.6%)	3.01	1	0.083
**Functioning** ^**b**^ (*N* = 362)								
Met needs	84 (23.2%)	295	68 (23.1%)	67	16 (23.9%)	0.02	1	0.885
Unmet needs	164 (45.3%)	295	131 (44.4%)	67	33 (49.3%)	0.52	1	0.472
**Health** ^**c**^ (*N* = 379)								
Met needs	71 (18.7%)	309	61 (19.7%)	70	10 (14.3%)	1.12	1	0.291
Unmet needs	284 (74.9%)	309	231 (74.8%)	70	53 (75.7%)	0.03	1	0.868
**Social** ^**d**^ (*N* = 375)								
Met needs	45 (12.0%)	305	38 (12.5%)	70	7 (10.0%)	0.33	1	0.568
Unmet needs	193 (51.5%)	305	153 (50.2%)	70	40 (57.1%)	1.11	1	0.292
**Services** ^**e**^ (*N* = 379)								
Met needs	97 (25.6%)	309	80 (25.9%)	70	17 (24.3%)	0.08	1	0.781
Unmet needs	118 (31.1%)	309	96 (31.1%)	70	22 (31.4%)	0.003	1	0.953

^a^Accommodation, Food, Day time activities

^b^Looking after home, Self-care, Child-care, Education, Money, Work

^c^Physical health, Psychotic symptoms, Psychological distress, Safety to self, Safety to others, Alcohol, Drugs

^d^Company, Intimate relationship, Sexual expression

^e^Telephone, Transport, Welfare benefits, Information

*Met need:* One or more met needs but no unmet needs on the domains within the dimension. *Unmet need:* At least one unmet need on the domains belonging to the dimension. *N, n* = number of participants, *X*^*2*^ = Chi^2^-value, *df* = degrees of freedom

## Discussion

The most remarkable result to emerge from the data was a higher overall satisfaction with the received mental health care treatment in the past 12 months among patients with migration background compared to those without. Simultaneously, no differences were shown in the utilization of treatments as well as in the degree of accordance between needed and actually received mental healthcare between patients with and without migratory background.

Our results on patient satisfaction are in agreement with a Canadian study, which provided evidence for a higher satisfaction with mental health care among 1st generation migrants compared to native Canadians [35]. In contrast to the present results, higher dissatisfaction among patients with migratory background was found by Parkman and colleagues (1997) [36] in a mental healthcare context as well as by Borde and colleagues (2002) [37] in a somatic-gynecological context. Generally speaking, so far only a limited number of comparative studies on patient satisfaction between patients with and without migratory background exist and results are inconsistent [13–15].

Against the background of inconsistent data and the weight of evidence that speaks for strong and persistent mental healthcare disparities due to ethnicity and migration (e.g., access to or quality of care) [38] – how can the higher overall satisfaction rates among migrant patients be explained?

To begin with, it is crucial to examine potential confounding effects of socio-demographic characteristics on the relationship between migration and treatment satisfaction [14, 16]. In our sample, participants with and without migratory background exhibited significant differences in their health status (functioning level and severity of mental disorder), diagnosis and geographic characteristics. *Health status:* There is consistent evidence for a relationship between poor health status and overall lower satisfaction levels, for literature reviews see Badri et al. (2009) [20] and Batbaatar et al. (2017) [14]. In the present study, participants with migration background were identified to exhibit a significantly lower functioning level as well as higher severity of mental disorder compared to non-migrants. This finding would imply a lower satisfaction level among migrants – according to previous research. However, migrants reported to be overall more satisfied with their treatment, despite their lower health status. *Diagnosis:* In our sample, the diagnosis schizophrenia (ICD-10: F2x) was more prevalent among migrant compared to non-migrant subjects. This is in line with previous findings on elevated risks among migrant groups to develop non-affective psychoses, see Fearon and Morgan (2005) for a review [39]. The potential confounding role of diagnosis on the relationship between migration and treatment satisfaction appears to be small – the majority of studies about the relationship between satisfaction and diagnosis found no [13] or inconsistent effects [40, 41]. *Geographic characteristics:* In the present study, subjects with migration background were more often residents of medium populated areas (20′001–500′000 inhabitants), whereas participants without migratory background inhabited lower populated areas (≤ 20′000 inhabitants) more frequently. No differences were found in highly populated areas (> 500′000 inhabitants). Research on the relationship between population density and treatment satisfaction show inconsistent results. Few studies showed a higher overall satisfaction among rural residents [42, 43], whereas the majority of studies reported no [44, 45] or varying differences between rural and urban populations in treatment satisfaction [46–48]. Thus, research assigns solely a minor role to geographical characteristics in explaining treatment satisfaction [48]. To conclude, the effects of health status, diagnosis and geographic characteristics on the revealed differences in treatment satisfaction between patients with and without migratory background remain unclear. Thus, future research must be undertaken to further clarify the significance of socio-demographic characteristics on treatment satisfaction among migrant populations.

Furthermore, differences in satisfaction can be attributed to disparities in the provision of treatments (e.g., treatment accessibility). Regulated by a nationwide framework for demand planning, the geographic distribution of services is based on aspects like number of inhabitants per physician or specific regional characteristics. However, particularly in outpatient mental healthcare, there is a wide range in the supply density. For instance, patients from higher populated areas (metropolitan areas or Western Germany) experience a higher density of outpatient psychiatric and psychotherapeutic services [49, 50]. According to that, it would be expectable to find a lower service utilization among participants without migration background as they were more often residents of lower populated areas (≤ 20′000 inhabitants). However, as mentioned above, no significant intergroup differences in the utilization of mental healthcare services were detected. Around two-thirds of migrants and non-migrants reported the use of individual psychotherapy and nearly 100% the use of medication in the past 12 months (including out- and the current inpatient setting at the time of the study). Likewise, no differences were found in outpatient psychological-psychotherapeutic and medical-psychiatric treatment 3 months before admission to the clinic. In both groups the utilization rate was one-fourth for outpatient psychological- and around one-third for outpatient medical-psychiatric treatments. Concerning psychosocial treatments, patients with and without migration background did not exhibit differences in the lifetime use – participants of both groups reported to have received about one-third of the presented psychosocial interventions. At first sight, our findings are not in line with recent findings, which indicate an underrepresentation of migrant patients in the German in- and outpatient mental healthcare system [7–9]. However, comparing the proportion of migrants in the overall German population (26%) [1] with the proportion of migrants in the study population (19%), an underrepresentation of people with a migration background in the participating inpatient health care facilities can be observed. Thus, it remains unclear to which degree the underrepresentation of migrant participants in our study affected our results.

Equivalent to the results on service use, no difference in the degree of accordance between needed and actually received mental healthcare was found between migrants and non-migrants. Likewise, the secondary analyses that were conducted did not show intergroup differences (see supplement). This indicates that migrants and non-migrants received treatment according to their needs to a comparable extent. Despite the significance of met and unmet needs in the theoretical construct of patient satisfaction [16, 51] and also existing evidence on the relationship between unmet needs and lower satisfaction [52], the explored differences on satisfaction in our sample cannot be (sufficiently) explained by the degree of met and unmet needs. Moreover, our results showed that the number of unmet needs exceeds the number of met needs in each category examined - for both groups. These findings differ from previous results reported in the literature – Swedish researchers found a higher total number of unmet needs among the migrant compared to the non-migrant group and a higher number of met needs compared to unmet needs for both groups [53]. However, to our knowledge, research on fulfillment of patient’s needs among migrant groups does not exist for a German population and is also internationally sparse. Hence, further research needs to be performed to investigate the role of fulfillment of needs in patient satisfaction among migrants in the mental healthcare system (e.g., with emphasis on culturally sensitive evaluation tools on patient’s needs).

Participants with vs. without migration background significantly differed in their *Overall Satisfaction* and only numerically in the remaining categories (except for *Information*). *Overall Satisfaction* is not composed of the remaining specific categories of satisfaction (e.g., *Professionals’ Skills*) but rather represents a superordinate and more general level of satisfaction assessed by three items: satisfaction with the amount of help received, the kind of treatment services and the overall treatment services. On the one hand, it would be plausible to assume, a higher overall level of satisfaction is also reflected in a higher satisfaction on specific aspects of treatment e.g., on the information provided about diagnosis and treatment forms (*Information*). On the other hand, our results highlight the complexity of the construct *treatment satisfaction*. Despite its popularity, there is uncertainty about the construct and its various dimensions based on patient’s expectations, needs and their actual experiences [16, 54]. Another possible explanation of the seemingly inconsistent results on treatment satisfaction (overall vs. specific) might be a lack of discriminatory power between the two compared samples (participants with vs. without migration background). When comparing participants born in Germany with 1st generation migrants in our sample, differences in treatment satisfaction expand – 1st generation migrants reported to be more satisfied with most of the presented categories (*Professionals’ Skills and Behavior, Efficacy, Relatives Involvement, and Overall Satisfaction) –* except for *Information and Access,* where we did not detect a significant difference (see Supplementary Table 6). First generation migrants exhibit – unlike 2nd generation migrants - direct migration experiences and are possibly more influenced by the sociocultural factors of the country of origin. Therefore, it is essential to investigate the detected differences in treatment satisfaction against the background of sociocultural factors as well as factors related to migration itself. Culture, defined as collective phenomenon that characterizes persons, which share a set of defining values, norms, and attitudes [55], has uncontestably an influence on people’s social behaviors - and thus possibly on the expression of dissatisfaction [56]. The cultural dimensions by Hofstede are a well-established framework to describe and compare cultures [57]. One of its central components is the dimension *individualism-collectivism*, which describes the level of integration into groups. Whereas in individualist cultures ties are rather lose and the individual plays the central role, in collectivist cultures the focus is on groups and tightly integrated social relationships [58]. Numerous studies have investigated the impact of individualism and collectivism on social interaction patterns [56]. It was found that collectivist – in contrast to individualist cultures - tend to avoid the expression of dissatisfaction in order to avert potential conflicts [59–61]. In our study, the specific country of origin was not assessed and must be considered as limitation (see below). However, based on the current migration report of the German government (as of 2021), the four most prevalent migration backgrounds (1st or 2nd generation) are Turkish (13%), Polish (11%), Russian (7%) and Kazakh (6%) [62]. Each of these nations are – according to Hofstede’s research [58] – collectivist societies, whereas Germany is categorized as predominately individualistic society. Although Hofstede’s cultural dimensions must be interpreted with caution due to vast methodological and conceptual limitations (e.g., lack of sample representativeness, underestimation of a nation’s heterogeneity, neglection of non-cultural causation) [63], possible cultural differences (individualism – collectivism) and thus the preparedness to express discontentment rather than avoiding it, might partly explain differences in self-reported treatment satisfaction between patients with and without migration background. Socially desirable behavior and therefore the expression of satisfaction is not only related to cultural aspects. Moreover, it was observed that the expression of satisfaction is also associated with migration itself [16, 63]. Recent qualitative research indicates a lack of expressed dissatisfaction in case of inadequate treatment services among patients with migration background [64, 65]. Language skills, obligation of gratitude and thus socially desirable behavior are discussed as possible influential factors on expressing or mitigating negative treatment experiences [16, 63–65]. Additionally, previous literature has discussed the possible impact of prior treatment expectations on treatment satisfaction [16]. It was considered that unsatisfying experiences with the healthcare system in the country of origin lead to a mitigation of negative treatment experiences and thus to an overestimation of satisfaction. However, according to the confirmation bias – the tendency to select, determine and interpret information in a manner that fulfils (confirms) one’s prior expectations – negative treatment experiences might rather lead to a decreased treatment satisfaction.

In conclusion, specific sociocultural (e.g., individualism vs. collectivism) as well as migration-related factors (e.g., language barriers, social desirability) might contribute to a higher actual or solely expressed satisfaction. In that regard, further research is needed to investigate the impact of migration and cultural norms on the perceived as well as expressed treatment satisfaction among migrant patients.

There are some limitations concerning the results of this study. As first limitation, results on migrant groups must be interpreted with caution. Migrant groups are highly heterogeneous and exhibit a great diversity of cultural, ethnic, religious and social backgrounds and therefore diverging experiences, attitudes and behaviors towards healthcare [22]. Moreover, distinction was made in our sample neither by country of origin nor by reason for migration. Given that the impact of both aspects could not be examined for the present research questions, results must thus be treated with caution. Furthermore, as we conducted our study in psychiatric and psychosomatic inpatient and day hospital facilities, the participants received intense and diverse treatment services. Therefore, the degree of satisfaction, which our participants exhibited, might not be generalizable to other settings (e.g., outpatient services) or to the general population. Also, our study did not include the assessment of needs identified by clinicians due to the focus on the patient’s perspective of the overall project. Therefore, data based on patients’ needs must be interpreted with regard to the missing comparative variable. Moreover, significant differences in geographic characteristics, health status and diagnosis between participants with and without migration background were detected. Given the differences, results must be interpreted with caution. The following limitation concerns the number of different variables. While we corrected post-hoc contrasts for multiple comparisons, we did not adjust analyses according to the total number of tested variables. Hence, the exploratory findings must be replicated in independent samples. Furthermore, the proportion of migrants in our sample (19%) does not correspond to the proportion of migrants in the German population as a whole (26%) [1]. Even though it was expectable due to the found underrepresentation of migrants in the health system [6], it can bias the present results as it does not capture the remaining 7%, which might have stayed away from the mental healthcare system due to dissatisfaction or were not included in the study due to language barriers. Finally, we are not able to make statements regarding those patients who did not want to participate or who stopped the trial early. Thus, a selection bias regarding those patients with a higher level of satisfaction cannot be completely ruled out. The same risk for a selection bias can be assumed by the inclusion criterion of having sufficient German language skills.

## Conclusion

To our knowledge, the present work is the first comparative multicenter study on satisfaction with mental healthcare services between migrant and non-migrant inpatients in German psychiatric facilities. Taken together, the present findings indicate a higher overall satisfaction with mental healthcare services among migrants. Simultaneously, no differences in service use as well as in met/unmet needs in mental healthcare were detected between patients with and without migratory background. Moreover, findings on satisfaction appear to be associated with sociocultural and migration-related factors – the explored differences on treatment satisfaction increased when comparing 1st generation migrants with native Germans (without migration background or 2nd generation migrants). In conclusion, the present work supports the significance of sociocultural and migration-related factors for (expression of) treatment satisfaction, e.g., social desirability. Thus, our findings point towards the risk to overlook the needs of patients with migration background in our healthcare system and highlight the importance of an exhaustive exploration of patients’ needs and expectations within a culturally sensitive healthcare setting. However, in order to understand the role of sociocultural and migrant-related factors on patient satisfaction and to provide more specific practical implications, further research must be undertaken.

## Supplementary Information

**Additional file 1.**

## Data Availability

The datasets generated and/or analysed during the current study are not publicly available due to limitations of ethical approval involving the patient data and anonymity but are available from the corresponding author on reasonable request.

## Footnotes

1 In the present article, we based our definition of migration/migratory background on the definition provided by the Federal Statistical Office of Germany [1]: A person has a migration background if he or she or at least one of his or her parents did not acquire the German citizenship at birth.
