# Supplementary material for: The role of migration in mental healthcare: treatment satisfaction and utilization
Source: BMC Psychiatry. 2022 Feb 15;22:116. doi: 10.1186/s12888-022-03722-8 (PMC8845273; doi:10.1186/s12888-022-03722-8)
Supplement: Supplementary file 1 — Additional file 1. [file 12888_2022_3722_MOESM1_ESM.docx]

**Supplement**

1. **Supplementary Methods**
   1. **Subjects and recruitment**

Data was collected in the following departments for psychiatry and psychotherapy in Upper Bavaria and Swabia: Department of Psychiatry and Psychotherapy at the University Hospital Munich, kbo-Isar-Amper-Hospital Munich-Haar, kbo-Isar-Amper-Hospital Munich-Schwabing, kbo-Isar-Amper-Hospital Taufkirchen, District Hospital Guenzburg, District Hospital Kempten, District Hospital Memmingen, District Hospital Donauwoerth, District Hospital Augsburg, and District Hospital Kaufbeuren. The selected centers represent metropolitan (Munich, Augsburg), middle-urban (Kempten, Memmingen) as well as rural (Donauwörth, Günzburg, Kaufbeuren, Taufkirchen) catchment areas. The questions were exclusively asked in German. As a parallel to that, in Germany the social health insurances in general do not pay for translation services [1]. However, most hospitals pay translational services by themselves, have contracts with telephone interpreting services or have a database of lay interpreter.

- 1. **Service satisfaction and utilization**

Conceptually, the 54 items of the VSSS-54 [2] represent seven dimensions: *Overall Satisfaction* (three items: satisfaction with the amount of help received, the kind of treatment services, the overall treatment services), *Professionals’ Skills and Behavior* (24 items: satisfaction with professionals’ behavior, e.g. interpersonal skills), *Information* (three items: satisfaction with information on disorders, therapies and services), *Access* (two items: satisfaction with service location and costs), *Efficacy* (eight items: satisfaction with overall and specific aspects of efficacy of service, e.g. social skills), *Relatives Involvement* (six items: satisfaction with help given to relatives/persons of trust) and *Types of Intervention* (17 items: satisfaction with and use of e.g. medical prescription, psychotherapy). The items of the VSSS-54 dimensions were rated on a 5-point Likert scale (level of satisfaction: 1 = terrible - 5 = excellent), the dimension *Types of Intervention,* however, consisted of binary on service utilization (“Did you receive the intervention in the last year?”) and categorial items on the evaluation of treatments received. In our study, we only conducted analyses of the dimension *Types of Intervention* regarding binary information about the use or of specific treatment interventions (Yes vs. No). Moreover, because of a hypothesis-based item selection and two missing items (item 53 and 54), the dimension *Types of Intervention* was not analyzed on a dimension-based but on an item-based level. The utilization of psychosocial interventions was surveyed with the questionnaire “Attitudes and knowledge regarding psychosocial therapies” developed by the authors and available on request. Against the background of hypothesis-based item selection, we analyzed the binary items on the utilization of the presented psychosocial therapy forms (e.g., question: “Have you ever received supported employment?”, answer: Yes/no). In our analyses, consideration was given to all categories of psychosocial interventions: *single psychosocial interventions* [8 items: Psychoeducation (general and specific), self-care support (e.g. personal hygiene), social skills training, arts therapies, occupational therapy, exercise- and movement therapy, health promoting interventions (e.g., nutrition, sport)], *cross-cutting issues* (6 items: Self-management, internet- or computer-based self-help, self-help manuals, self-help groups, counselling and support by peers, trialogues/psychosis seminars) and *system-level interventions* (11 items: Assertive community treatment, multidisciplinary home treatment, case management, outpatient residential care interventions, inpatient residential care interventions, family-based residential care interventions, work training under protected conditions, supported employment, supporting measures in the workplace, support to maintain employment, supported employment during studies/training/education). To quantify the utilization of psychosocial interventions, we computed a percentual score, which corresponds to the percentage of psychosocial interventions used out of the total number of psychosocial interventions presented.

- 1. **Patient needs**

The interviewer-administered instrument CAN-EU [3] originally consists of 23 individual domains of need which can be summarized to the following five categories of need: *Basic* (accommodation, food and daytime activities), *Health* (physical health, psychotic symptoms, psychological distress, safety to self, safety to others, alcohol and drugs), *Social* (company, intimate relationships and sexual expression), *Functioning* (looking after the home, self-care, childcare, education, money and work) and *Services* (information, telephone, transport and benefits).

1. **Supplementary Results – Secondary analyses**

We conducted the following secondary analyses: First, we computed a three-group-comparison as sensitivity analysis, identifying differences between 1^st^ generation migrants, 2^nd^ generation migrants and subjects without migratory background. Second, we analyzed contrasts between native Germans (subjects without migratory background and 2^nd^ generation migrants) and 1^st^ generation migrants (two-group comparison).

- 1. **Sensitivity analysis: Three-group comparison**

| Supplementary Table 1. Descriptive statistics and mean response comparisons. | | | | | | | | | | | | | | | | | | | | | | | |  |
| --- | --- | --- | --- | --- | --- | --- | --- | --- | --- | --- | --- | --- | --- | --- | --- | --- | --- | --- | --- | --- | --- | --- | --- | --- |
|  |  | | | | |  | | | | |  | | | | | **Test Statistics** | | | | | | | |  |
|  | **No migration**  **background (No)** | | | | | **2^nd^ generation migrants (2^nd^)** | | | | | **1^st^ generation**  **migrants (1^st^)** | | | | |  | |  | | |  | | |  |
|  | **N** | | **n (% Yes)** | | | **N** | | **n (% Yes)** | | | **N** | | **n (% Yes)** | | | **X²** | | **df** | | | **p** | | |  |
| **Gender** (*N* = 387) |  |  | | |  | |  | | | | |  | |  | | |  | | |  | | |  |  |
| Female | 315 | | 137 (43.5%) | | | 23 | | 9 (39.1%) | | | 49 | | 21 (42.9%) | | | 0.17 | | 2 | | | 0.919 | | |  |
| **Diagnosis ^a^** (*N* = 387) |  | | | | | | | | | | | | | | | | | | | | | | |  |
| Schizophrenia | 315 | | 94 (29.8%) | | | 23 | | 11 (47.8%) | | | 49 | | 20 (40.8%) | | | 6.68 | | | 4 | | | 0.154 | |  |
| Depression | 315 | | 187 (59.4%) | | | 23 | | 11 (47.8%) | | | 49 | | 27 (55.1%) | | |  |  |  |  |  |  |  |  |  |
| Bipolar Disorder | 315 | | 34 (10.8%) | | | 23 | | 1 (4.3%) | | | 49 | | 2 (4.1%) | | |  |  |  |  |  |  |  |  |  |
| **Family status** (*N* = 387) |  | | | | | | | | | | | | | | | | | | | | | | |  |
| Single | 315 | | 185 (58.7%) | | | 23 | | 17 (73.9%) | | | 49 | | 15 (30.6%) | | | 21.73 | | 6 | | | **0.001** | | |  |
| Married | 315 | | 65 (20.6%) | | | 23 | | 6 (26.1%) | | | 49 | | 17 (34.7%) | | |  |  |  |  |  |  |  |  |  |
| Divorced | 315 | | 56 (17.8%) | | | 23 | | 0 (0.0%) | | | 49 | | 13 (26.5%) | | |  |  |  |  |  |  |  |  |  |
| Widowed | 315 | | 9 (2.9%) | | | 23 | | 0 (0.0%) | | | 49 | | 4 (8.2%) | | |  |  |  |  |  |  |  |  |  |
| **Population size** (*N* = 386) |  | | | | | | | | | | | | | | | | | | | | | | |  |
| ≤ 20 000 | 314 | | 143 (45.5%) | | | 23 | | 4 (17.4%) | | | 49 | | 13 (26.5%) | | | 14.16 | | | 4 | | | **0.007** | |  |
| 20 001 – 500 000 | 314 | | 92 (29.3%) | | | 23 | | 13 (56.5%) | | | 49 | | 20 (40.8%) | | |  |  |  |  |  |  |  |  |  |
| > 500 000 | 314 | | 79 (25.2%) | | | 23 | | 6 (26.1%) | | | 49 | | 16 (32.7%) | | |  |  |  |  |  |  |  |  |  |
|  | | | | | | | | | | | | | | | | | | | | | | | |  |
|  | **N** | | **M (SD)** | | | **N** | | **M (SD)** | | | **N** | | **M (SD)** | | | **F** | | **df** | | | **p** | | |  |
| **Age** (*N* = 385) |  | | | | | | | | | | | | | | | | | | | | | | |  |
| Years | 313 | | 43.16  (13.14) | | | 23 | | 36.22  (14.39) | | | 49 | | 43.96  (11.42) | | | 3.25 | | 2 | | | **0.040** | | |  |
| **Salary** (*N* = 151) |  | | | | | | | | | | | | | | | | | | | | | | |  |
| Euro, net | 130 | | 1686.37  (1125.65) | | | 5 | | 1122.00  (1087.99) | | | 16 | | 1543.75  (1070.96) | | | 0.70 | | 2 | | | 0.500 | | |  |
| **GAF ^b^** (*N* = 387) |  | | | | | | | | | | | | | | | | | | | | | | |  |
|  | 315 | | 42.92 (9.91) | | | 23 | | 36.26 (7.38) | | | 49 | | 41.08 (8.96) | | | 5.53 | | 2 | | | **0.004** | | |  |
| **HoNOS ^c^** (*N* = 387) |  | | | | | | | | | | | | | | | | | | | | | | |  |
|  | 315 | | 22.03 (5.97) | | | 23 | | 24.00 (5.61) | | | 49 | | 23.43 (5.85) | | | 2.16 | | 2 | | | **0.117** | | |  |
|  |  | |  | | |  | |  | | |  | |  | | |  | |  | | |  | | |  |
|  |  | |  | | |  | |  | | |  | |  | | |  | |  | | |  | | |  |
|  | **Subgroup analyses: Chi Square Test**  **(*p*-values Bonferroni corrected)** | | | | | | | | | | | | | | |  | | | | | | | | |
|  | **No _[vs]_ 2^nd^** | | | | | **No _[vs]_ 1^st^** | | | | **1^st^ _[vs]_ 2^nd^** | | | | | |  | | | | | | | | |
|  | **X²** | | | **p** | | **X²** | | | **p** | **X²** | | | | | **p** |  | | | | | | | | |
| **Family status** |  | | |  | |  | | |  |  | | | | |  |  | | | | | | | | |
| Single | 2.06 | | | 0.456 | | 13.54 | | | **0.001** | 11.89 | | | | | **0.003** |  | | | | | | | | |
| Married | 0.38 | | | 1.000 | | 4.80 | | | 0.084 | 0.53 | | | | | 1.000 |  | | | | | | | | |
| Divorced | 4.90 | | | 0.081 | | 2.12 | | | 0.438 | 7.45 | | | | | **0.018** |  | | | | | | | | |
| Widowed | 0.68 | | | 1.000 | | 3.47 | | | 0.100 | 1.99 | | | | | 0.477 |  | | | | | | | | |
| **Population size** |  | | |  | |  | | |  |  | | | | |  |  | | | | | | | | |
| ≤ 20 000 | 6.91 | | | **0.027** | | 6.25 | | | **0.036** | 0.73 | | | | | 1.000 |  | | | | | | | | |
| 20 001 – 500 000 | 7.40 | | | **0.021** | | 2.64 | | | 0.315 | 1.56 | | | | | 0.636 |  | | | | | | | | |
| > 500 000 | 0.01 | | | 1.000 | | 1.23 | | | 0.801 | 0.32 | | | | | 1.000 |  | | | | | | | | |
|  |  | | |  | |  | | |  |  | | | | |  |  | | | | | | | | |
|  | **Subgroup analyses: Bonferroni Test**  **(*p*-values Bonferroni corrected)** | | | | | | | | | | | | | | |  | |  | | |  | | |  |
|  |  |  |  |  |  |  |  |  |  |  |  |  |  |  |  |  | |  | | |  | | |  |
|  | **No _[vs]_ 2^nd^** | | | | | **No _[vs]_ 1^st^** | | | | | **1^st^ _[vs]_ 2^nd^** | | | | |  | |  | | |  | | |  |
|  | **[I-J]** | | | | | **[I-J]** | | | | | **[I-J]** | | | | |  | |  | | |  | | |  |
|  | **95% CI** | | | | | **95% CI** | | | | | **95% CI** | | | | |  | |  | | |  | | |  |
|  | **p** | | | | | **p** | | | | | **p** | | | | |  | |  | | |  | | |  |
| **Age** (Years) |  | | | | |  | | | | |  | | | | |  | |  | | |  | | |  |
|  | 6.94 | | | | | -0.80 | | | | | 7.74 | | | | |  | |  | | |  | | |  |
|  | [0.18; 13.70] | | | | | [-5.61; 4.00] | | | | | [-0.17; 15.65] | | | | |  | |  | | |  | | |  |
|  | **0.042** | | | | | 1.000 | | | | | 0.057 | | | | |  | |  | | |  | | |  |
| **GAF ^b^** |  | | | | |  | | | | |  | | | | |  | |  | | |  | | |  |
|  | 6.66 | | | | | 1.84 | | | | | 4.82 | | | | |  | |  | | |  | | |  |
|  | [1.64;11.68] | | | | | [-1.73; 5.41] | | | | | [-1.06; 10.70] | | | | |  | |  | | |  | | |  |
|  | **0.005** | | | | | 0.649 | | | | | 0.148 | | | | |  | |  | | |  | | |  |
|  |  | |  | | |  | |  | | |  | |  | | |  | |  | | |  | | |  |
| *Note.* ^a^The diagnosis assignment was based on the ICD-10 classification system: F2x (schizophrenia), F32, F33 (depression), F30, F31 (bipolar disorder). ^b^Global Assessment of Functioning: higher values indicate a higher level of functioning. ^c^Health of the Nation Outcome Scales: higher values indicate a higher severity of mental disorder *N* = number of participants, *M* = means, *SD* = standard deviations, *X²* = Chi²-value, *F* = F-statistics, *df* = degrees of freedom, *I-J* = difference in mean between groups. *95% CI* = 95% confidence interval for the difference in means. | | | | | | | | | | | | | | | | | | | | | | | |  |

| Supplementary Table 2. Treatment satisfaction: Average confirmation rates of the VSSS-EU dimensions (Verona Service Satisfaction Scale – European Version) and response comparisons. | | | | | | | | | | | | | | | | | | | | |
| --- | --- | --- | --- | --- | --- | --- | --- | --- | --- | --- | --- | --- | --- | --- | --- | --- | --- | --- | --- | --- |
|  |  | | |  | |  | | |  | | |  | | |  | |  | **KWT** | | |
|  | **No migration**  **background (No)** | | | | | **2^nd^ generation migrants (2^nd^)** | | | | | | **1^st^ generation**  **migrants (1^st^)** | | | | |  |  |  |  |
|  | **N** | **Mdn** | **M** | | **SD** | **N** | **Mdn** | **M** | | **SD** | **N** | | **Mdn** | **M** | | **SD** |  | **H** | **df** | **p** |
| **Overall Satisfaction** | 298 | 3.67 | 3.72 | | 0.80 | 23 | 4.00 | 4.00 | | 0.60 | 45 | | 4.00 | 4.01 | | 0.87 |  | 7.44 | 2 | **0.024** |
| **Professionals’ Skills and Behavior** | 144 | 4.00 | 3.88 | | 0.71 | 10 | 4.03 | 4.05 | | 0.58 | 15 | | 4.13 | 4.28 | | 0.44 |  | 4.41 | 2 | 0.110 |
| **Information** | 285 | 3.67 | 3.38 | | 0.95 | 23 | 3.67 | 3.48 | | 0.95 | 38 | | 3.67 | 3.65 | | 0.88 |  | 2.98 | 2 | 0.226 |
| **Access** | 276 | 3.50 | 3.57 | | 0.92 | 21 | 4.00 | 3.86 | | 1.20 | 44 | | 4.00 | 3.75 | | 0.74 |  | 3.34 | 2 | 0.188 |
| **Efficacy** | 140 | 3.50 | 3.29 | | 0.91 | 11 | 3.38 | 3.51 | | 0.96 | 16 | | 3.88 | 3.80 | | 0.94 |  | 4.34 | 2 | 0.114 |
| **Relatives Involvement** | 125 | 3.40 | 3.21 | | 1.14 | 7 | 4.20 | 4.09 | | 0.58 | 15 | | 4.40 | 3.95 | | 1.18 |  | 10.62 | 2 | **0.005** |
|  |  |  |  | |  |  |  |  | |  |  | |  |  | |  |  |  |  |  |
|  | **Subgroup analyses: Dunn-Bonferroni-Test**  **(*p*-values Bonferroni corrected)** | | | | | | | | | | | | | | | |  |  |  |  |
|  | **No _[vs]_ 2^nd^** | | | | | **No _[vs]_ 1^st^** | | | | | **1^st^ _[vs]_ 2^nd^** | | | | | |  |  |  |  |
|  | **Z** | | **p** | | | **Z** | | **p** | | | **Z** | | | **p** | | |  |  |  |  |
| **Overall Satisfaction** | 1.60 | | 0.332 | | | 2.36 | | 0.055 | | | 0.12 | | | 1.000 | | |  |  |  |  |
| **Relatives Involvement** | 2.09 | | 0.111 | | | 2.65 | | **0.024** | | | -0.19 | | | 1.000 | | |  |  |  |  |
| *Note.* The items of the presented dimensions were rated on a 5-point Likert scale (level of satisfaction: 1 = terrible - 5 = excellent). *N* = number of participants, *Mdn* = medians, *M* = means, *SD* = standard deviations, *KWT* = Kruskal-Wallis test, *H =* H-value, *df* = degrees of freedom, *Z* = standard score. | | | | | | | | | | | | | | | | | | | | |

| Supplementary Table 3. Service utilization: Average confirmation rates of the utilization of treatment and response comparisons. | | | | | | | | | | | | | | | | | | |
| --- | --- | --- | --- | --- | --- | --- | --- | --- | --- | --- | --- | --- | --- | --- | --- | --- | --- | --- |
|  |  |  | | |  |  | | | |  | |  | | |  | **Chi² Test** | | |
|  | **No migration**  **background (No)** | | | | **2^nd^ generation migrants (2^nd^)** | | | | | **1^st^ generation**  **migrants (1^st^)** | | | | |  |  |  |  |
|  | **N** | **n (% Yes)** | | | **N** | **n (% Yes)** | | | | **N** | | **n (% Yes)** | | |  | **X²** | **df** | **p** |
| **Medical treatment** |  |  | | |  |  | | | |  | |  | | |  |  |  |  |
| Medication prescription ^a^ | 294 | 290 (98.6%) | | | 22 | 20 (90.9%) | | | | 44 | | 43 (97.7%) | | |  | 6.44 | 2 | **0.040** |
| Outpatient medical-psychiatric treatment ^b^ | 312 | 127 (40.7%) | | | 23 | 9 (39.1%) | | | | 48 | | 15 (31.3%) | | |  | 1.56 | 2 | 0.459 |
| **Psychotherapeutic treatment** |  |  | | |  |  | | | |  | |  | | |  |  |  |  |
| Individual  psychotherapy ^a^ | 301 | 210 (69.8%) | | | 22 | 14 (63.6%) | | | | 47 | | 30 (63.8%) | | |  | 0.94 | 2 | 0.625 |
| Family therapy ^a^ | 299 | 64 (21.4%) | | | 22 | 5 (22.7%) | | | | 45 | | 4 (8.9%) | | |  | 3.95 | 2 | 0.139 |
| Outpatient psychological-psychotherapeutic treatment ^b^ | 312 | 72 (23.1%) | | | 23 | 8 (34.8%) | | | | 49 | | 9 (18.4%) | | |  | 2.38 | 2 | 0.304 |
|  |  |  | | |  |  | | | |  | |  | | |  |  |  |  |
|  |  |  | | |  |  | | | |  | |  | | |  | **KWT** | | |
|  | **N** | **Mdn** | | **M (SD)** | **N** | **Mdn** | | **M (SD)** | | **N** | | **Mdn** | | **M (SD)** |  | **H** | **df** | **p** |
| **Psychosocial treatment** |  |  | |  |  |  | |  | |  | |  | |  |  |  |  |  |
| Score (per cent) ^c^ | 314 | 0.28 | | 0.32 (0.17) | 23 | 0.28 | | | 0.28 (0.15) | | 47 | 0.24 | | 0.29 (0.15) |  | 1.89 | 2 | 0.389 |
|  |  |  | |  |  |  | | |  | |  |  | |  |  |  |  |  |
|  | **Subgroup analyses: Chi Square Test**  **(*p*-values Bonferroni corrected)** | | | | | | | | | | | | | |  |  |  |  |
|  | **No _[vs]_ 2^nd^** | | | | **No _[vs]_ 1^st^** | | | | | | **1^st^ _[vs]_ 2^nd^** | | | |  |  |  |  |
|  | **X²** | | **p** | | **X²** | | **p** | | | | **X²** | | **p** | |  |  |  |  |
| **Medication prescription ^a^** | 6.57 | | **0.030** | | 0.22 | | 1.000 | | | | 1.57 | | 0.630 | |  |  |  |  |
| *Note.* ^a^Participants were asked whether they had received the intervention in the last 12 months (VSSS-EU). The related time period of 12 months refers to the inpatient setting (at the time of the survey) and previous settings (out- or inpatient settings). ^b^Participants were asked whether they had received the intervention 3 months before admission to clinic (CSSRI-EU). ^c^Participants were asked whether they had ever received the psychosocial intervention. The displayed score corresponds to the percentage of psychosocial interventions used out of the total number of psychosocial interventions presented. *N* = number of participants, *X²* = Chi²-value, *df* = degrees of freedom, *Mdn* = medians, *M* = means, *SD* = standard deviations, *KWT* = Kruskal-Wallis test, *H =* H-value, *df* = degrees of freedom. | | | | | | | | | | | | | | | | | | |

| Supplementary Table 4. Met and unmet needs: Average confirmation rates of the CAN-EU dimensions of need (Camberwell Assessment of Need – European Version) and response comparisons. | | | | | | | | | | |
| --- | --- | --- | --- | --- | --- | --- | --- | --- | --- | --- |
|  |  | |  | |  | |  | **Chi² Test** | | |
|  | **No migration**  **background (No)** | | **2^nd^ generation migrants (2^nd^)** | | **1^st^ generation**  **migrants (1^st^)** | |  |  | | |
|  | **N** | **n (% Yes)** | **N** | **n (% Yes)** | **N** | **n (% Yes)** |  | **X²** | **df** | **p** |
| **Basic ^a^** (*N* = 378) |  |  |  |  |  |  |  |  |  |  |
| Met needs | 308 | 84 (27.3%) | 23 | 3 (13.0%) | 47 | 9 (19.1%) |  | 3.39 | 2 | 0.183 |
| Unmet needs | 308 | 115 (37.3%) | 23 | 12 (52.2%) | 47 | 22 (46.8%) |  | 3.20 | 2 | 0.202 |
| **Functioning ^b^** (*N* = 362) |  |  |  |  |  |  |  |  |  |  |
| Met needs | 295 | 68 (23.1%) | 23 | 5 (21.7%) | 44 | 11 (25.0%) |  | 0.11 | 2 | 0.946 |
| Unmet needs | 295 | 131 (44.0%) | 23 | 11 (47.8%) | 44 | 22 (50.0%) |  | 0.55 | 2 | 0.761 |
| **Health ^c^** (*N* = 379) |  |  |  |  |  |  |  |  |  |  |
| Met needs | 309 | 61 (19.7%) | 23 | 4 (17.4%) | 47 | 6 (12.8%) |  | 1.33 | 2 | 0.514 |
| Unmet needs | 309 | 231 (74.8%) | 23 | 16 (69.6%) | 47 | 37 (78.7%) |  | 0.72 | 2 | 0.699 |
| **Social ^d^** (*N* = 375) |  |  |  |  |  |  |  |  |  |  |
| Met needs | 305 | 38 (12.5%) | 23 | 1 (4.3%) | 47 | 6 (12.8%) |  | 1.36 | 2 | 0.506 |
| Unmet needs | 305 | 153 (50.2%) | 23 | 14 (60.9%) | 47 | 26 (55.3%) |  | 1.30 | 2 | 0.522 |
| **Services ^e^** (*N* = 379) |  |  |  |  |  |  |  |  |  |  |
| Met needs | 309 | 80 (25.9%) | 47 | 3 (13.0%) | 23 | 14 (29.8%) |  | 2.35 | 2 | 0.309 |
| Unmet needs | 309 | 96 (31.1%) | 47 | 11 (47.8%) | 23 | 11 (23.4%) |  | 4.30 | 2 | 0.117 |
| *Note.* ^a^Accommodation, Food, Day time activities ^b^Looking after home, Self-care, Child-care, Education, Money, Work. ^c^Physical health, Psychotic symptoms, Psychological distress, Safety to self, Safety to others, Alcohol, Drugs. ^d^Company, Intimate relationship, Sexual expression. ^e^Telephone, Transport, Welfare benefits, Information. *Met need:* One or more met needs but no unmet needs on the domains within the dimension. *Unmet need:* At least one unmet need on the domains belonging to the dimension. *N* = number of participants, *X²* = Chi²-value, *df* = degrees of freedom. | | | | | | | | | | |

- 1. **Sensitivity analysis: Two-group comparison**

| Supplementary Table 5. Descriptive statistics and mean response comparisons. | | | | | | | | | | | | | | | | | | |
| --- | --- | --- | --- | --- | --- | --- | --- | --- | --- | --- | --- | --- | --- | --- | --- | --- | --- | --- |
|  | |  | | | | | |  | | | | | | | **Test Statistics** | | | |
|  | | **No or 2^nd^ generation migrants** | | | | | | **1^st^ generation**  **migrants** | | | | | | |  |  | |  |
|  | | **N** | | **n (% Yes)** | | | | **N** | | **n (% Yes)** | | | | | **X²** | **df** | | **p** |
| **Gender** (*N* = 387) | |  | |  | | | |  | |  | | | | |  | | | |
| Female | | 338 | | 192 (56.8%) | | | | 49 | | 28 (57.1%) | | | | | 0.002 | 1 | | 0.964 |
| **Diagnosis ^a^** (*N* = 387) | |  | |  | | | |  | |  | | | | |  | | | |
| Schizophrenia | | 338 | | 105 (31.1%) | | | | 49 | | 20 (40.8%) | | | | | 3.11 | | 2 | 0.211 |
| Depression | | 338 | | 198 (58.6%) | | | | 49 | | 27 (55.1%) | | | | |  |  |  |  |
| Bipolar Disorder | | 338 | | 35 (10.4%) | | | | 49 | | 2 (4.1%) | | | | |  |  |  |  |
| **Family status** (*N* = 387) | |  | |  | | | |  | |  | | | | |  | | | |
| Single | | 338 | | 202 (59.8%) | | | | 49 | | 15 (30.6%) | | | | | 14.77 | | 1 | **<0.001** |
| Married | | 338 | | 71 (21.0%) | | | | 49 | | 17 (34.7%) | | | | | 4.56 | | 1 | **0.033** |
| Divorced | | 338 | | 56 (16.6%) | | | | 49 | | 13 (26.5%) | | | | | 2.90 | | 1 | 0.089 |
| Widowed | | 338 | | 9 (2.7%) | | | | 49 | | 4 (8.2%) | | | | | 3.99 | | 1 | **0.046** |
| **Population size** (*N* = 386) | |  | |  | | | |  | |  | | | | |  | | | |
| ≤ 20 000 | | 337 | | 147 (43.6%) | | | | 49 | | 13 (26.5%) | | | | | 5.15 | | 2 | 0.076 |
| 20 001 – 500 000 | | 337 | | 105 (31.2%) | | | | 49 | | 20 (40.8%) | | | | |  |  |  |  |
| > 500 000 | | 337 | | 85 (25.2%) | | | | 49 | | 16 (32.7%) | | | | |  |  |  |  |
|  | |  | |  | | | |  | |  | | | | | | | | |
|  | | **N** | | **M (SD)** | | | | **N** | | **M (SD)** | | | | | **t** | **df** | | **p** |
| **Age** (*N* = 385) | | | | | | | | | | | | | | | | | | |
| Years | | 336 | | 42.68 (13.32) | | | | 49 | | 43.96 (11.42) | | | | | -0.64 | 383 | | 0.524 |
| **Salary** (*N* = 151) | | | | | | | | | | | | | | | | | | |
| Euro, net | | 135 | | 1665.47 (1125.43) | | | | 16 | | 1543.75 (1070.96) | | | | | 0.41 | 149 | | 0.682 |
| **GAF ^b^** (*N* = 387) | | | | | | | | | | | | | | | | | | |
|  | | 338 | | 42.47 (9.89) | | | | 49 | | 41.08 (8.96) | | | | | 0.93 | 385 | | 0.355 |
| **HoNOS ^c^** (*N* = 387) | | | | | | | | | | | | | | | | | | |
|  | | 338 | | 22.16 (5.95) | | | | 49 | | 23.43 (5.85) | | | | | -1.39 | 385 | | 0.690 |
| *Note.* ^a^The diagnosis assignment was based on the ICD-10 classification system: F2x (schizophrenia), F32, F33 (depression), F30, F31 (bipolar disorder). ^b^Global Assessment of Functioning: higher values indicate a higher level of functioning. ^c^Health of the Nation Outcome Scales: higher values indicate a higher severity of mental disorder *N* = number of participants, *M* = means, *SD* = standard deviations, *X²* = Chi²-value, *t* = t-statistics, *df* = degrees of freedom. | | | | | | | | | | | | | | | | | | |
| Supplementary Table 6. Treatment satisfaction: Average confirmation rates of the VSSS-EU dimensions (Verona Service Satisfaction Scale – European Version) and response comparisons. | | | | | | | | | | | | | | | | | | |
|  |  | | | |  | |  | | | |  | |  | **MWU Test** | | | | |
|  | **No or 2^nd^ generation migrants** | | | | | | **1^st^ generation**  **migrants** | | | | | |  |  | |  | |  |
|  | **N** | | **Mdn** | | **M** | **SD** | **N** | | **Mdn** | | **M** | **SD** |  | **U** | | **Z** | | **p** |
| **Overall Satisfaction** | 321 | | 3.67 | | 3.74 | 0.79 | 45 | | 4.00 | | 4.01 | 0.87 |  | 5764.50 | | -2.23 | | **0.027** |
| **Professionals’ Skills and Behavior** | 154 | | 4.00 | | 3.89 | 0.70 | 15 | | 4.13 | | 4.28 | 0.44 |  | 788.50 | | -2.03 | | **0.043** |
| **Information** | 308 | | 3.67 | | 3.39 | 0.95 | 38 | | 3.67 | | 3.65 | 0.88 |  | 4880.00 | | -1.68 | | 0.093 |
| **Access** | 297 | | 4.00 | | 3.59 | 0.94 | 44 | | 4.00 | | 3.75 | 0.74 |  | 6080.50 | | -0.75 | | 0.451 |
| **Efficacy** | 151 | | 3.38 | | 3.31 | 0.91 | 16 | | 3.88 | | 3.80 | 0.94 |  | 834.50 | | -2.03 | | **0.042** |
| **Relatives Involvement** | 132 | | 3.40 | | 3.26 | 1.13 | 15 | | 4.40 | | 3.95 | 1.18 |  | 599.50 | | -2.50 | | **0.012** |
| *Note.* The items of the presented dimensions were rated on a 5-point Likert scale (level of satisfaction: 1 = terrible - 5 = excellent). *N* = number of participants, *Mdn* = medians, *M* = means, *SD* = standard deviations, *MWU* = Mann-Whitney-U Test, *U* = U-value, *Z* = standard score. | | | | | | | | | | | | | | | | | | |

| Supplementary Table 7. Service utilization: Average confirmation rates of the utilization of treatment and response comparisons. | | | | | | | | | | | |
| --- | --- | --- | --- | --- | --- | --- | --- | --- | --- | --- | --- |
|  |  |  | |  |  | |  | **Chi² Test** | | |  |
|  | **No or 2^nd^ generation migrants** | | | **1^st^ generation**  **migrants** | | |  |  |  |  |  |
|  | **N** | **n (% Yes)** | | **N** | **n (% Yes)** | |  | **X²** | **df** | **p** |  |
| **Medical treatment** |  |  | |  |  | |  |  |  |  |  |
| Medication prescription ^a^ | 316 | 310 (98.1%) | | 44 | 43 (97.7%) | |  | 0.03 | 1 | 0.866 |  |
| Outpatient medical-psychiatric treatment ^b^ | 312 | 127 (40.7%) | | 71 | 24 (33.8%) | |  | 1.15 | 1 | 0.283 |  |
| **Psychotherapeutic treatment** |  |  | |  |  | |  |  |  |  |  |
| Individual psychotherapy ^a^ | 323 | 224 (69.3%) | | 47 | 30 (63.8%) | |  | 0.58 | 1 | 0.446 |  |
| Family therapy ^a^ | 321 | 69 (21.5%) | | 45 | 4 (8.9%) | |  | 3.93 | 1 | **0.047** |  |
| Outpatient psychological-psychotherapeutic treatment ^b^ | 312 | 72 (23.1%) | | 72 | 17 (23.6%) | |  | 0.01 | 1 | 0.923 |  |
|  |  |  | |  |  | |  |  |  |  |  |
|  |  |  | |  |  | |  | **MWU Test** | | |  |
|  | **N** | **Mdn** | **M**  **(SD)** | **N** | **Mdn** | **M (SD)** |  | **U** | **Z** | **p** |  |
| **Psychosocial treatment** |  |  |  |  |  |  |  |  |  |  |  |
| Score (per cent) ^c^ | 337 | 0.28 | 0.31 (0.17) | 47 | 0.24 | 0.29 (0.15) |  | 7138.50 | -1.10 | 0.272 |  |
| *Note.* ^a^Participants were asked whether they had received the intervention in the last 12 months (VSSS-EU). The related time period of 12 months refers to the inpatient setting (at the time of the survey) and previous settings (out- or inpatient settings). ^b^Participants were asked whether they had received the intervention 3 months before admission to clinic (CSSRI-EU). ^c^Participants were asked whether they had ever received the psychosocial intervention. The displayed score corresponds to the percentage of psychosocial interventions used out of the total number of psychosocial interventions presented. *N* = number of participants, *X²* = Chi²-value, *df* = degrees of freedom, *Mdn* = medians, *M* = means, *SD* = standard deviations, *MWU* = Mann-Whitney-U Test, *U* = U-value, *Z* = standard score | | | | | | | | | | | |

| Supplementary Table 8. Met and unmet needs: Average confirmation rates of the CAN-EU dimensions of need (Camberwell Assessment of Need – European Version) and response comparisons. | | | | | | | | |
| --- | --- | --- | --- | --- | --- | --- | --- | --- |
|  |  | |  | |  | **Chi² Test** | | |
|  | **No or 2^nd^ generation migrants** | | **1^st^ generation**  **migrants** | |  |  | | |
|  | **N** | **n (% Yes)** | **N** | **n (% Yes)** |  | **X²** | **df** | **p** |
| **Basic ^a^** |  |  |  |  |  |  |  |  |
| Met needs | 331 | 87 (26.3%) | 47 | 9 (19.1%) |  | 1.11 | 1 | 0.293 |
| Unmet needs | 331 | 127 (38.4%) | 47 | 22 (46.8%) |  | 1.23 | 1 | 0.268 |
| **Functioning ^b^** |  |  |  |  |  |  |  |  |
| Met needs | 318 | 73 (23.0%) | 44 | 11 (25.0%) |  | 0.09 | 1 | 0.763 |
| Unmet needs | 318 | 142 (44.7%) | 44 | 22 (50.0%) |  | 0.45 | 1 | 0.504 |
| **Health ^c^** |  |  |  |  |  |  |  |  |
| Met needs | 332 | 65 (19.6%) | 47 | 6 (12.8%) |  | 1.26 | 1 | 0.263 |
| Unmet needs | 332 | 247 (74.6%) | 47 | 37 (78.7%) |  | 0.41 | 1 | 0.522 |
| **Social ^d^** |  |  |  |  |  |  |  |  |
| Met needs | 328 | 39 (11.9%) | 47 | 6 (12.8%) |  | 0.03 | 1 | 0.863 |
| Unmet needs | 328 | 167 (50.9%) | 47 | 26 (55.3%) |  | 0.32 | 1 | 0.572 |
| **Services ^e^** |  |  |  |  |  |  |  |  |
| Met needs | 332 | 83 (25.0%) | 47 | 14 (29.8%) |  | 0.50 | 1 | 0.481 |
| Unmet needs | 332 | 107 (32.2%) | 47 | 11 (23.4%) |  | 1.50 | 1 | 0.221 |
| *Note.* ^a^Accommodation, Food, Day time activities ^b^Looking after home, Self-care, Child-care, Education, Money, Work. ^c^Physical health, Psychotic symptoms, Psychological distress, Safety to self, Safety to others, Alcohol, Drugs. ^d^Company, Intimate relationship, Sexual expression. ^e^Telephone, Transport, Welfare benefits, Information. *Met need:* One or more met needs but no unmet needs on the domains within the dimension. *Unmet need:* At least one unmet need on the domains belonging to the dimension. *N* = number of participants, *X²* = Chi²-value, *df* = degrees of freedom. | | | | | | | | |

1. **References**

1. Wissenschaftliche Dienste des Deutschen Bundestages. Dolmetscher im Rahmen der gesundheitlichen Versorgung Anspruch und Kostenübernahme Sachstand Wissenschaftliche Dienste. 2017. https://www.bundestag.de/resource/blob/514142/d03782888dd292a2ed12cffd271d8ecb/wd-9-021-17-pdf-data.pdf. Accessed 12 Nov 2021.

2. Mory C, Matschinger H, Roick C, Kilian R, Bernert S, Angermeyer MC. Die deutsche Version der Verona Service Satisfaction Scale (VSSS-54) - Ein Instrument zur Erfassung der Patientenzufriedenheit mit psychiatrischer Behandlung -. Psychiatr Prax. 2001;28:91–6. doi:10.1055/s-2001-17791.

3. Kilian R, Bernert S, Matschinger H, Mory C, Roick C, Angermeyer MC. Die standardisierte Erfassung des Behandlungs- und Unterstützungsbedarfs bei schweren psychischen Erkrankungen: Entwicklung und Erprobung der deutschsprachigen Version des Camberwell Assessment of Need-EU. Psychiatr Praxis, Suppl. 2001;28:79–83. doi:10.1055/s-2001-17789.
